# Novel Antimicrobial Surfaces to Defeat COVID-19 Transmission

**DOI:** 10.1557/adv.2020.418

**Published:** 2020-12-01

**Authors:** Rodica Cristescu, Roger J. Narayan, Douglas B. Chrisey

**Affiliations:** 11 lasma & Radiation Physics, Lasers Department, National Institute for Lasers, Bucharest-Magurele, Romania; 21 Biomedical Engineering, University of North Carolina, Chapel Hill, NC USA; 31 Department of Physics and Engineering Physics, Tulane University, New Orleans, LA USA

## Abstract

Antimicrobial surface coatings function as a contact biocide and are extensively used to prevent the growth and transmission of pathogens on environmental surfaces. Currently, scientists and researchers are intensively working to develop antimicrobial, antiviral coating solutions that would efficiently impede/stop the contagion of COVID-19 via surface contamination. Herein we present a flavonoid-based antimicrobial surface coating fabricated by laser processing that has the potential to eradicate COVID-19 contact transmission. Quercetin-containing coatings showed better resistance to microbial colonization than antibiotic–containing ones.

## INTRODUCTION:

The coronavirus disease 2019 (COVID-19) pandemic continues to disrupt everyday life worldwide. The number of confirmed cases is increasing, and although countries are gradually opening, there is a constant threat of a second wave [1]. On September 17th, 2020, the virus had infected over 30.2 million people globally, with around 940,000 reported casualties [2]. The global health outbreak generated by COVID-19 has urged the search for preventive and therapeutic treatments for this pathogen; the severe acute respiratory syndrome coronavirus 2 (SARS-CoV-2). Many efforts and resources have been dedicated to patient management, better diagnosis, improving protective care, decreasing organ failure cases, reducing drug side effects, and developing a preventive vaccine. Even if the vaccine development is promising and imminent, efficient drugs are still compulsory for infected people. The transmission of this deadly virus occurs through direct contact with infected people and indirect contact with contaminated surfaces. It is critical to find a solution that can prevent the transmission of the virus present on surfaces. An antimicrobial, antiviral surface coating could effectively inhibit bacterial adhesion and subsequent biofilm formation. The coating thus reduces the risk of environmental infections. On the other hand, the cost-efficient drugs are of the essence for reaching all the population [3]. In this respect, an important screening study has been focused on the identification of an inhibitor of SARS-COV-2 3CLpro, the main protease that plays an essential role in the virus replication cycle by processing the large viral polyproteins and providing the functionality of the individual protein. From a total of about 150 compounds, a natural flavonoid product, namely quercetin, has been designated as SARS-COV-2 3CLpro inhibitor with suitable potency (inhibition constant Ki ~ 7 μM) to be promoted as a good candidate for further optimization and development in preclinical studies [4]. It has been also demonstrated that quercetin would be an appropriate scaffold to engineer new functional groups for developing new inhibitor compounds for SARS-CoV-2 3CLpro. The best therapeutic approach to minimize the probability of drug resistance occurrence consists of a combination of several drugs that act through different mechanisms [5-7]. Our studies established a novel approach to fabricate antimicrobial surface composite coatings and evaluated their *in vitro* biological performance. These composite coatings can be deposited by a wide variety of techniques that range in their complexity and applicability. The selection of an appropriate deposition technique depends upon the physicochemical properties of the composite material, requirements for film quality, and substrate that is being coated. The goal of our studies has been to obtain thin, uniform, and adherent coatings over an extended substrate surface area and in localized areas. We aim to surmount this drawback by using laser light for processing due to its advantages: accurate and precise thickness control, preservation of their chemical integrity, physicochemical properties desired, compatibility with non-contact masking techniques, and minimum possible degradation. Among the laser processing techniques, the most suitable approach to fabricate high quality assays appropriate for antimicrobial investigations is Matrix Assisted Pulsed Laser Evaporation (MAPLE) technology [8]. The patented MAPLE method can fabricate thin films and coatings of high quality, uniform, and adherent polymeric, organic, biomaterial, and composite materials on various substrates [9]. These coatings are grown with accurate controlled thickness ranging from 5 nm to several micrometers without affecting the structure, chemical properties, or biological activity of the deposited material. In MAPLE, a frozen matrix comprising a polymer, pharmaceutical, or other organic material in a relatively volatile matrix is used as laser target. The type of matrix and appropriate concentration are chosen so that the solute, in our case composite material, can be dissolved to form a dilute solution. Then, the solution is frozen by immersion in liquid nitrogen (77 K) and kept in direct contact with a cooler. This way, the rapid target evaporation is significantly slowed down. In MAPLE, the used incident laser pulse generates two photothermal processes in the matrix: (i) evaporating the frozen material target (nanocomposite, in our case), and (ii) releasing the composite nanomaterial into the chamber. Due to the low concentration of nanocomposite material in the target (1-5) %, the simultaneous action of the evaporation softly desorbs the composite material. The photon energy absorbed by the matrix is converted to thermal energy that causes the complex material heating and matrix vaporization. After this, composite material molecules are exposed at the gas-target matrix interface. The nanocomposite material molecules achieve sufficient kinetic energy through collective collisions with the evaporating matrix molecules to be transferred in the gas phase. By careful optimization of the MAPLE deposition conditions (laser wavelength, repetition rate, matrix type, concentration, target-substrate distance, target and substrate rotation speed values, substrate temperature, type and pressure of background gas), this process can occur without any major nanomaterial decomposition. The MAPLE process proceeds layer-by-layer, depleting the target of matrix and nanocomposite material compound in the same concentration as the initial matrix. The MAPLE set-up used in experiments is depicted schematically in Fig. 1.

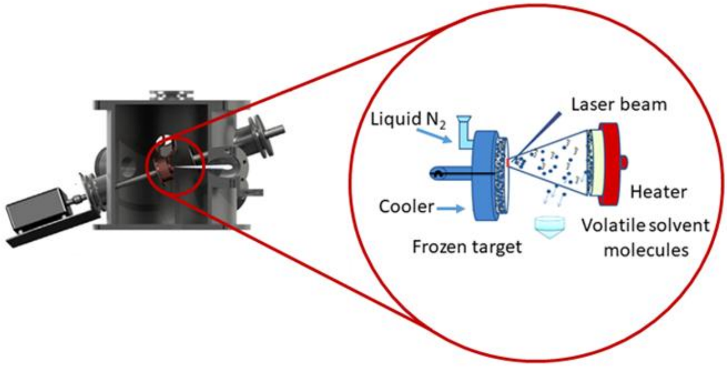

**Fig. 1** MAPLE experimental setup.

The precise thickness control at the nanometric scale produces a better control of drug content, which is crucial since the synthesis of sufficient quantities of an antimicrobial coating is challenging and expensive [10]. Extension of MAPLE to composite materials is interesting, owing to its reliability and good control over deposition process parameters. This way, MAPLE technique offers an effective way to integrate novel antimicrobial functionality into complex nanocomposite-based thin films that are difficult, if not impossible, to achieve otherwise.

## EXPERIMENTAL DETAILS:

In our MAPLE experiments, we have chosen to combine the following categories of antimicrobial compounds: *conventional large-spectrum antibiotics* (norfloxacin and cefuroxime), *systemic antifungal agents* (amphotericin B and voriconazole), *unconventional bioactive organic* (vegetal flavonoids compounds: quercetin dihydrate and resveratrol), as well as *inorganic molecules* (silver nanoparticles). These active compounds will be embedded in polyvinylpyrrolidone *biopolymer* and assembled in *composite forms*. Thus, functionalized nanostructured composite coatings, specifically biopolymer - antibiotic, biopolymer - flavonoid, and biopolymer - flavonoid - antibiotic have been synthesized as thin coatings deposited using MAPLE. The solvent used for MAPLE target preparation was dimethyl sulfoxide (DMSO). Thin composite coatings were deposited by MAPLE and drop-casting. Before each deposition, 3.5 mL of the newly prepared solution was placed using a syringe in a pre-cooled copper target holder that had a 3 cm diameter and a 5 mm height. The MAPLE cryogenic target was created by placing the target in a Dewar vessel that contains liquid nitrogen. After the freezing step, the target holder was quickly placed in the target position within the matrix-assisted pulsed laser evaporation chamber. The MAPLE thin films were obtained onto optical glass slides and one-side polished Si <100> wafers. To get clean and sterilized substrates, we used both an ethanol ultrasonic bath and a UV lamp. A KrF* excimer laser source ($\lambda$ = 248 nm@10 Hz, 25 ns pulse duration) that was operated at a fluence of 50-500 mJ/cm$^2$ a repetition rate of 10 Hz, and for 10,000-140,000 pulses was utilized for MAPLE deposition. The laser beam browsed the target surface at a 45° angle. A laser beam homogenizer was utilized to enhance the distribution of energy for the laser spot and increase the coated region on the substrate. During MAPLE experiments, the target and substrate were placed at 5 cm separation distance; both were rotated at a rate of 0.4 Hz. All of the depositions were conducted with a background pressure of (0.1-0.5) Pa and a substrate-to-target distance of (4-5) cm. The rotating target was kept in direct contact with a cooling device that contained a liquid nitrogen reservoir; it was connected to the target using copper pipes. Rapid evaporation of MAPLE target inside the deposition chamber is reduced using this setup.

## RESULTS AND DISCUSSION:

In a *first attempt*, in view of obtaining improved coatings capable of modulating and controlling microbial biofilm behavior, we used MAPLE to fabricate thin composite coatings resistant to microbial colonization, containing natural (**quercetin flavonoid**) and synthetic (**norfloxacin** and **cefuroxime** antibiotics) compounds [11]. The optimum laser fluence for which we obtained high quality MAPLE-deposited composite coatings in terms of surface uniformity (a uniform surface formed by globular structures) and structural integrity preservation has been identified (300 mJ/cm$^2$) [11]. Both the anti-biofilm efficiency of norfloxacin (**Nor**) and cefuroxime (**Cef**) antimicrobial agents embedded in the polyvinylpyrrolidone (**PVP**) biopolymer and prospective simultaneous activity of quercetin (**Q**) with the investigated antimicrobial agents have been evaluated. The anti-biofilm assays have been performed on two bacterial strains typical for Gram-positive (*Staphylococcus aureus* (*S. aureus* ATCC 25923)) and Gram-negative (*Pseudomonas aeruginosa* (*P. aeruginosa* ATCC 27853*)*) microorganisms that are usually involved in the aetiology of biofilm-related infections. Microbial biofilm growth on optical glass slides coated with the experimental synthesis variants has been evaluated at 48 and 72 h, respectively. In the case of *S. aureus* biofilms (Fig. 2), the utmost number of microbial cells in biofilm has been registered at 48 h, while the number of biofilms–enclosed viable cells considerably diminished at 72 h. This outcome could be assigned to the choice of a persisting population, represented by microbial cells that were viable, but were not developed in the vicinity of the inhibitory agent. The polyvinylpyrrolidone (**PVP**) biopolymer itself has not presented any substantial antimicrobial activity in comparison to the uncoated optical glass slide ($p > 0.05$). The most effective antimicrobial combination against *S. aureus* biofilms of 48 h proved by far to be polyvinylpyrrolidone – norfloxacin (**PVP-Nor**) (generating a 9 log decreasing of colony-forming unit (CFU)number) ($p < 0.001$), followed by polyvinylpyrrolidone – cefuroxime (**PVP-Cef**) ($p < 0.001$)**,** polyvinylpyrrolidone – quercetin – norfloxacin (**PVP-Q-Nor**) ($p < 0.001$), quercetin – norfloxacin (**Q-Nor**) ($p < 0.001$) and quercetin – cefuroxime **(Q-Cef**) ($p < 0.001$), revealing a 4 orders of magnitude decrease of biofilm embedded cells. At 72 h, under different experimental conditions, the biofilm reduction has been far lower (1-2 logs), while the most effective combinations have been confirmed to be polyvinylpyrrolidone – cefuroxime (**PVP-Cef),** quercetin – cefuroxime (**Q-Cef),** and polyvinylpyrrolidone – quercetin (**PVP-Q**) ($p < 0.001$). It has to be pointed out that polyvinylpyrrolidone – quercetin (**PVP-Q**) has showed a very good anti-biofilm activity, much superior to the scrutinized antimicrobial agents.

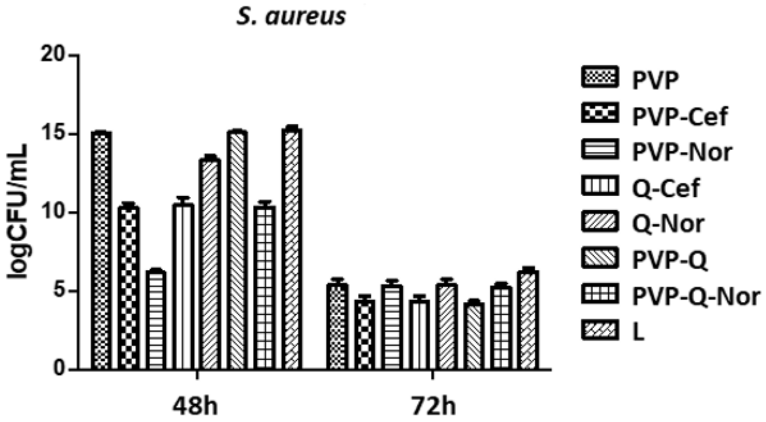

**Fig. 2**. Development of *S. aureus* biofilm on uncoated and coated glass slides. Negative control – sterile optical glass slides; polyvinylpyrrolidone (**PVP**); polyvinylpyrrolidone – – cefuroxime (**PVP-Cef**); polyvinylpyrrolidone – norfloxacin (**PVP-Nor**), quercetin – cefuroxime (**Q-Cef),** quercetin – norfloxacin (**Q-Nor**), polyvinylpyrrolidone – quercetin dihydrate (**PVP-Q**); polyvinylpyrrolidone – quercetin – norfloxacin (**PVP-Q-Nor**).

The dynamics of the *P. aeruginosa* biofilm has been distinct from that corresponding to *S. aureus* biofilm; the number of biofilm-enclosed cells continued to be relatively unchanged at 48 and 72 h, respectively (Fig. 3). It should be remarked that the polyvinylpyrrolidone (**PVP**) substrate alone facilitates bacterial biofilm proliferation at 48 h; yet, in combination with antibiotics, it ensures the antibiotic delivery in the active form, as shown by the decrease of the number of viable cells evaluated from CFU/ml data ($p < 0.001$). Like the case of *S. aureus* biofilms, polyvinylpyrrolidone – quercetin (**PVP-Q**) exhibited an anti-biofilm activity that is comparable to that of polyvinylpyrrolidone – quercetin – norfloxacin (**PVP-Q-Nor**) ($p < 0.001$), proving once more the anti-biofilm activity of quercetin. The result has been even more obvious in the case of 72 h biofilms, where the combination of quercetin with both antibiotics and polyvinylpyrrolidone radically diminished the number of viable cells within the biofilm ($p < 0.001$).

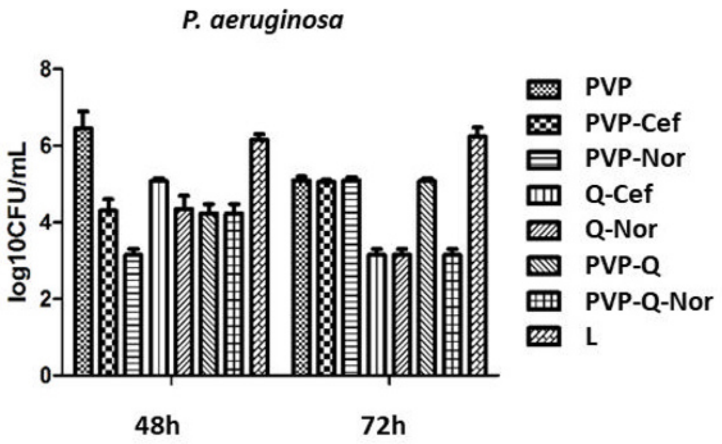

**Fig. 3.** Development of *P. aeruginosa* biofilm on uncoated and coated glass slides. Polyvinylpyrrolidone (**PVP**); polyvinylpyrrolidone – – cefuroxime (**PVP-Cef**); polyvinylpyrrolidone – norfloxacin (**PVP-Nor**), quercetin – cefuroxime (**Q-Cef**), quercetin – norfloxacin (**Q-Nor**), polyvinylpyrrolidone – quercetin dihydrate (**PVP-Q**); polyvinylpyrrolidone – quercetin – norfloxacin (**PVP-Q-Nor**). Negative control – sterile glass slides.

*Going further with our studies*, we have used MAPLE to fabricate *quercetin dihydrate* (**Q**)-, *resveratrol* (**R**)-, and *silver nanoparticle (**AgNP**)-polyvinylpyrrolidone (**PVP**) biopolymer* thin films, characterized their chemical structure and morphology, and evaluated their antimicrobial activity against both Gram-positive (*Staphylococcus aureus* (*S. aureus*, ATCC 29213)) and Gram-negative (*Escherichia coli* (*E. coli*, ATCC 25922)) bacteria in view of the potential use of these combination systems for the development of novel antimicrobial approaches [12]. Gentamicin was used as positive control. The silver nanoparticle (**AgNP**) was dispersed in $H_2O$ or ethylene glycol (EG). The structural and morphological investigations revealed that the best quality coatings (chemical structure with a very close resemblance to that of the initial (drop-cast) materials and surface morphology with AgNPs homogeneously dispersed into the coating) have been obtained at the ~80 mJ/cm$^2$ optimum laser fluence value [12]. In order to enhance the activity of the anti-bacterial surfaces, a combination of contact-based (the inhibition of bacterial growth at the contact surface with the MAPLE-coated glass slide) and release-based (inhibition region occurred around the MAPLE-coated glass slide) bactericidal effect, followed by the withdrawal of treated bacteria would be beneficial to design [13]. In this research, the modified disk diffusion assay has been used to explore the contact- and release-based bactericidal effect of the MAPLE-deposited coatings, respectively.

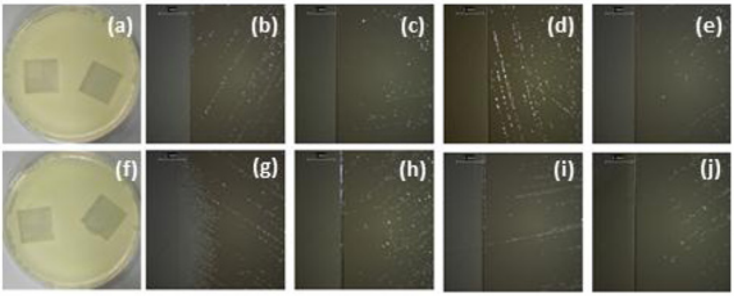

**Fig. 4.** Modified disk diffusion images of samples plated with *S. aureus*: (a) macroscopic view of negative control, (b) negative control, (c) polyvinylpyrrolidone, (d) polyvinylpyrrolidone – quercetin, (e) polyvinylpyrrolidone – resveratrol, (f) macroscopic view of positive control, (g) positive control, (i) polyvinylpyrrolidone – silver nanoparticles dispersed in $H_2O$, and (j) polyvinylpyrrolidone – silver nanoparticles dispersed in ethylene glycol.

The experimental data have been achieved through measuring the regions of growth inhibition for the microbial cultures to the nearest millimeter. Images of the plated samples are visible in Figs. 4-5. The positive controls that contained gentamicin caused the incidence of growth inhibition regions in the range of (19 - 23) mm for *S. aureus,* and (18 - 22) mm for *E. coli* test plates, respectively. Although a growth inhibition has been observed on the culture medium surface placed in direct contact with the MAPLE-coated optical glass slide, the regions of growth inhibition surrounding the MAPLE-coated samples were not noticeable. These results indicate a greater significant contact-induced growth inhibition, and a fewer efficient release-killing effect.

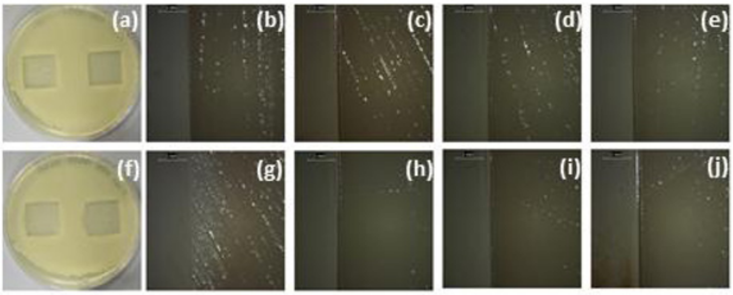

**Fig. 5.** Modified disk diffusion images of samples plated with *E. coli*: (a) macroscopic view of negative control, (b) negative control, (c polyvinylpyrrolidone, (d) polyvinylpyrrolidone – quercetin, (e) polyvinylpyrrolidone – resveratrol, (f) macroscopic view of positive control, (g) positive control, (i) polyvinylpyrrolidone – silver nanoparticles dispersed in $H_2O$, and (j) polyvinylpyrrolidone – silver nanoparticles dispersed in ethylene glycol.

The comparative study of the growth inhibition diameters has revealed that the most efficient antimicrobial combinations have been the films containing polyvinylpyrrolidone **(PVP)** and silver nanoparticles (**AgNPs**), and particularly polyvinylpyrrolidone – silver nanoparticles dispersed in ethylene glycol (**PVP-AgNP-EG**) (Fig. 6). In the case of *S. aureus* strain, the antibacterial activity of polyvinylpyrrolidone alone containing films has been similar or even higher than that of the polyvinylpyrrolidone - flavonoids combinations. These results could be correlated to the anti-fouling activity of the polyvinylpyrrolidone biopolymer that is able to confer a hydrophilic microenvironment, inhibiting the initial bacterial adherence that is mediated by the hydrophobic interactions with the substrate [14, 15]. Additionally, the preservation of a hydrophilic environment could promote the activity of other antibacterials comprised within the thin films (such as silver nanoparticles). Polyvinylpyrrolidone – quercetin (**PVP-Q**) thin films have demonstrated to be more effective against the *E. coli* strain, while polyvinylpyrrolidone – resveratrol (**PVP-R**) thin films showed activity against *S. aureus* (Fig. 6).

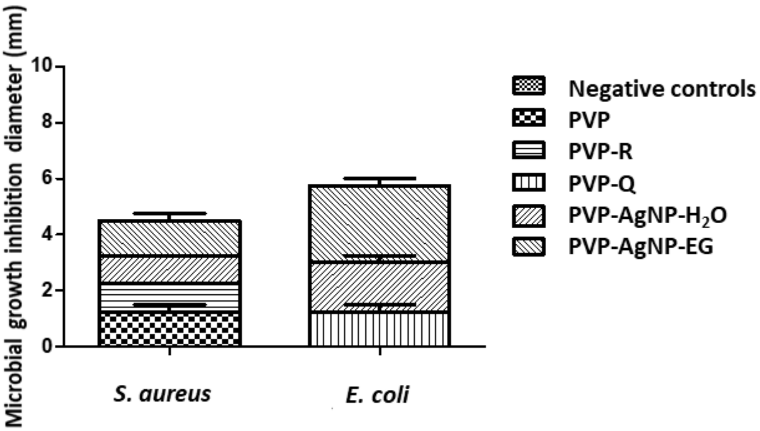

**Fig. 6.** Graphical representation of the antimicrobial activity of different MAPLE-coated glass slides. Negative control – sterile optical glass slides; polyvinylpyrrolidone (**PVP**); polyvinylpyrrolidone – resveratrol (**PVP-R**); polyvinylpyrrolidone – quercetin dihydrate (**PVP-Q**); polyvinylpyrrolidone – silver nanoparticles dispersed in $H_2O$ (**PVP-AgNP-$H_2O$**), and (j) polyvinylpyrrolidone – silver nanoparticles dispersed in ethylene glycol (**PVP-AgNP-EG**). Positive control data are not shown.

*In the latest study*, we used MAPLE to prepare different biomimetic thin films containing the polyvinylpyrrolidone (**PVP**) biopolymer, flavonoids (quercetin dihydrate (**Q**) and resveratrol (**R**)) and/or systemic antifungal agents (amphotericin B (**AmphB**) and voriconazole (**Vor**)). The chemical structures and morphologies of thin films were examined. The antifungal activity of thin films against two yeast strains*, Candida albicans (C. albicans*, ATCC 90028) and *Candida parapsilosis* (*C. parapsilosis*, ATCC 22019), was investigated to assess the potential of these materials for the development of novel antimicrobial strategies [16]. The chemical structure studies have proved that high-quality thin films with chemical structures similar to the drop-cast ones were MAPLE-coated using an optimum laser fluence of ~ 80 mJ/cm$^2$. The surface morphology investigations demonstrated that there are no grain boundaries. Also, no large material agglomerates were observed in the scanned samples [16]. In order to explore the fungal cultures that have been grown in contact with MAPLE-coated thin films, a slight adjustment of the disk diffusion assay was performed. Regions of growth inhibition in the fungal cultures are characteristic of the antifungal activity of the assessed materials; the diameters of the regions were measured to the nearest millimeter, as previously described. Images of the investigated samples are provided in Figs. 7 and 8, while the diameter values of the growth inhibition regions are presented in Fig. 9.

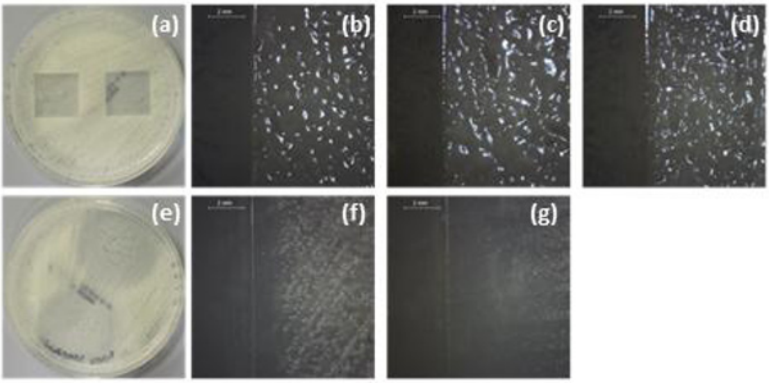

**Fig. 7.** Modified disk diffusion images of samples plated with C. *parapsilosis*: (a) macroscopic plate view of negative control, (b) negative control, (c) polyvinylpyrrolidone, (d) polyvinylpyrrolidone – amphotericin B, (e) macroscopic plate view of positive control, (f) positive control, and (g) polyvinylpyrrolidone – voriconazole.

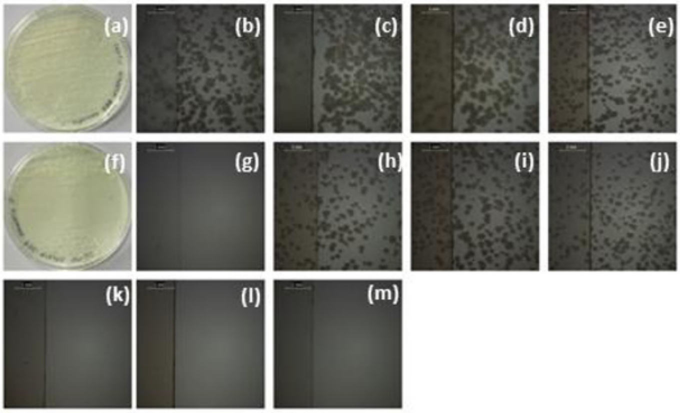

**Fig. 8.** Modified disk diffusion images of samples plated with *C. albicans*: (a) macroscopic view of negative control, (b) negative control, (c) polyvinylpyrrolidone, (d) polyvinylpyrrolidone – quercetin dihydrate, (e) polyvinylpyrrolidone – resveratrol, (f) macroscopic view of positive control, (g) positive control, (h) polyvinylpyrrolidone – quercetin dihydrate – amphotericin B, (i) polyvinylpyrrolidone – resveratrol – amphotericin B, (j) polyvinylpyrrolidone – amphotericin B, (k) polyvinylpyrrolidone – voriconazole, (l) polyvinylpyrrolidone – quercetin dihydrate – voriconazole, and (m) polyvinylpyrrolidone – resveratrol - voriconazole.

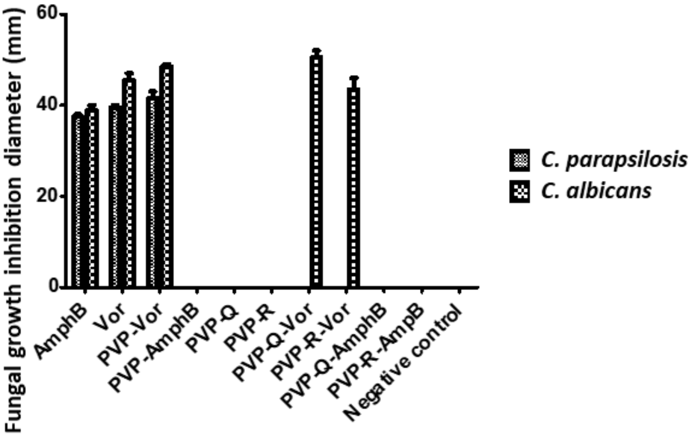

**Fig. 9**. Comparative representation of fungal growth inhibition diameters obtained in the presence of different thin composite coatings. Positive control - amphotericin B (**AmphB**), voriconazole (**Vor**); polyvinylpyrrolidone – voriconazole (**PVP-Vor**); polyvinylpyrrolidone – amphotericin B (**PVP-AmphB**); polyvinylpyrrolidone – quercetin dihydrate (**PVP-Q**); polyvinylpyrrolidone – resveratrol (**PVP-R**); polyvinylpyrrolidone – resveratrol - voriconazole (**PVP-Q-Vor**); polyvinylpyrrolidone – quercetin dihydrate – amphotericin B (**PVP-R-Vor**); polyvinylpyrrolidone – quercetin dihydrate – amphotericin B (**PVP-Q-AmphB**); - polyvinylpyrrolidone -resveratrol - amphotericin B (**PVP-R-AmphB**); Negative control – sterile optical glass slides.

In this research, the capacity of our biomimetic coatings to impede the growth of biofilms developed by *C. parapsilosis* and *C. albicans,* two fungal species frequently medical device-related infections, has been assessed under *in vitro* conditions. The achieved results have been substantially different for the two strains ($p < 0.001$). In the case of *C. parapsilosis* (Figs. 7 and 9), regions of growth inhibition have been examined for the positive controls (voriconazole (**Vor**), amphotericin B (**AmphB**)) and polyvinylpyrrolidone – voriconazole (**PVP-Vor**) MAPLE-coated samples. The incidence of growth inhibition regions suggests that these materials exhibit antifungal activity against *C. parapsilosis*, revealing an absence of detectable colony forming units (CFUs) in the regions surrounding the samples. This perception signifies that the antifungal activity of corresponding samples was not restricted to a surface interaction between the MAPLE-deposited coatings and fungal cells. The anti-biofilm activity of the coatings tested against *C. albicans* biofilms is visible in Fig. 8. The growth inhibition regions surrounding the samples were observed for the positive controls, polyvinylpyrrolidone - voriconazole (**PVP-Vor**), polyvinylpyrrolidone – quercetin dihydrate - voriconazole (**PVP-Q-Vor**), and polyvinylpyrrolidone – resveratrol – voriconazole (**PVP-R-Vor**) samples (Fig. 9). The analysis of the coating efficacy against *C. albicans* versus *C. parapsilosis* biofilms suggests a statistically significant higher activity of voriconazole (**Vor**), polyvinylpyrrolidone – voriconazole (**PVP-Vor**), polyvinylpyrrolidone – quercetin dihydrate – voriconazole (**PVP-Q-Vor**), and polyvinylpyrrolidone – resveratrol –

voriconazole (**PVP-R-Vor**) samples against *C. albicans* biofilms when compared to *C. parapsilosis* ones ($p < 0.001$). The coating consisting of voriconazole enclosed in polyvinylpyrrolidone (**PVP-Vor**) and polyvinylpyrrolidone – quercetin dihydrate (**PVP-Q-Vor**) proved to be substantially more active than the voriconazole (**Vor**) itself against *C. albicans* biofilms ($p < 0.05$ and $p < 0.0001$, respectively). The coatings comprising both voriconazole and quercetin dihydrate have revealed a higher activity against *C. albicans* biofilms, as compared to the coatings consisting of either voriconazole or quercetin dihydrate alone ($p < 0.001$). For *C. parapsilosis* biofilms, the coatings that incorporate polyvinylpyrrolidone-voriconazole (**PVP-Vor**) have been found more active than the voriconazole (**Vor**) alone ($p <0.001$).

It is noteworthy that our findings are in good accordance with the literature that validate the synergistic combination therapy of polyvinylpyrrolidone or quercetin and azoles. For instance, the quercetin combined with fluconazole has evidenced their promising synergistic activity in the clinical management of vulvovaginal candidiasis caused by *C. albicans* biofilms [17]. In other case, the voriconazole pills may contain excipients such as polyvinylpyrrolidone biopolymer as granulation binder [18]. Other relevant studies have been focused on demonstrating the efficacy of combinational use of polyvinylpyrrolidone-coated silver nanoparticles and conventional azole antifungals (e.g., fluconazole, voriconazole) to combat the CA10 drug-resistant *C. albicans* strain infection [19].

As a whole, our results highlight that the polyvinylpyrrolidone biopolymer constitutes an interesting biomimetic matrix for targeted and controlled local release of antifungal voriconazole, but not for the delivery of amphotericin B or flavonoid bioactive compounds of plant origin taken apart. This drug release strategy could enhance the therapeutic efficiency and reduce the systemic toxic effects of voriconazole.

## CONCLUSIONS:

The presented work provides a direct evidence for quercetin ability to target SARS-CoV-2 3CLpro main protease. Quercetin, a natural product with well-known pharmacokinetics, exhibits antioxidant, antiallergic, anti-inflammatory, and anti-proliferative indications. Most important, quercetin can be directed for drug repositioning, which points to a potential repurposing for COVID-19 treatment. We have demonstrated that the MAPLE technique is an appropriate approach for obtaining biomimetic thin films containing combinations of antimicrobial compounds: *conventional large-spectrum antibiotics* (norfloxacin and cefuroxime), *systemic antifungal agents* (amphotericin B and voriconazole), *unconventional bioactive organic* (vegetal flavonoids compounds: quercetin dihydrate and resveratrol), as well as *inorganic molecules* (silver nanoparticles) that exhibit chemical structures with a very close resemblance to that of the initial materials and a uniform surface morphology. Agar diffusion assays has validated the antifungal activity of polyvinylpyrrolidone-voriconazole and polyvinylpyrrolidone – quercetin dihydrate - voriconazole containing MAPLE-coated films against *C. parapsilosis* and *C. albicans*, respectively. These findings suggest the potential application of polyvinylpyrrolidone biopolymer and quercetin flavonoid combination for advanced voriconazole delivery systems that both enhance the therapeutic efficacy of the antifungal agent at the site of infection and reduce the side effects of voriconazole. Also, coatings containing both voriconazole and quercetin have revealed an improved efficiency against *C. albicans* biofilms. The thin films fabricated by MAPLE have shown antibacterial efficiency against Gram-positive and Gram-negative bacterial strains, substantially mediated by a contact-killing effect, demonstrating the potential application of these composite systems and deposition approach for the development of innovative, safe, green antimicrobial strategies. We emphasize that the quercetin used in our work exhibits a significant anti-biofilm activity similar to that of the investigated large-spectrum antibiotics, in particular for the case of 72 h biofilms. These results could be important for the possible use of quercetin as a reliable substitute to antibiotics to inhibit mature biofilm growth on various substrates.

A medical device containing these MAPLE-deposited thin films may be evaluated by the US Food & Drug Administration as a combination product since the device includes features of devices and drugs [20]. The US Food & Drug Administration organized an Office of Combination Products in 2002 to enable suitable regulation of combination devices. The complexity of the approval process for a given combination device is governed by the regulatory status of the constituent drug(s) and device(s). The Investigational Device Exemption (IDE) process rules the regulatory processes for combination products that are supervised as medical devices and the Investigational New Drug (IND) process manages the regulatory processes for combination products that are regulated as drugs.

It is also important to note that the commercial production of the MAPLE thin film is required to follow the Good Manufacturing Practice (GMP) regulation of the US Food, Drug, and Cosmetic Act, which requires that medical devices complete specific quality assurance tasks, including (a) written manufacturing processes that confirm that manufacturing of the medical device matches that of the original design, (b) specifications for labelling of the medical device, (c) written protocols for process controls, (d) quality assurance activities, as well as (e) labelling specifications [21, 22].

## ACKNOWLEDGMENTS:

R.C. acknowledges the financial support of the Romanian National Authority for Scientific Research and Innovation, CNCS UEFISCDI, project no. PN-III-P4-ID-PCE-2016-0884 (142/2017, BIOMATE) within PNCDI III, and NUCLEU Programme – 16N/2019.
